# Microscale Self-Assembly of Upconversion Nanoparticles Driven by Block Copolymer

**DOI:** 10.3389/fchem.2020.00836

**Published:** 2020-09-16

**Authors:** Qianqian Su, Meng-Tao Zhou, Ming-Zhu Zhou, Qiang Sun, Taotao Ai, Yan Su

**Affiliations:** ^1^Institute of Nanochemistry and Nanobiology, Shanghai University, Shanghai, China; ^2^Department of Chemistry, National University of Singapore, Singapore, Singapore; ^3^Center for Functional Materials, NUS (Suzhou) Research Institute, Suzhou, China; ^4^National and Local Joint Engineering Laboratory for Slag Comprehensive Utilization and Environmental Technology, School of Materials Science and Engineering, Shaanxi University of Technology, Hanzhong, China; ^5^Genome Institute of Singapore, Agency of Science Technology and Research, Singapore, Singapore

**Keywords:** lanthanide-doped nanoparticles, upconversion nanoparticle, self-assembly, micro-scale, nanoparticles belt

## Abstract

Lanthanide-based upconversion nanoparticles can convert low-energy excitation to high-energy emission. The self-assembled upconversion nanoparticles with unique structures have considerable promise in sensors and optical devices due to intriguing properties. However, the assembly of isotropic nanocrystals into anisotropic structures is a fundamental challenge caused by the difficulty in controlling interparticle interactions. Herein, we report a novel approach for the preparation of the chain-like assemblies of upconversion nanoparticles at different scales from nano-scale to micro-scale. The dimension of chain-like assembly can be fine-tuned using various incubation times. Our study observed Y-junction aggregate morphology due to the flexible nature of amphiphilic block copolymer. Furthermore, the prepared nanoparticle assemblies of upconversion nanoparticles with lengths up to several micrometers can serve as novel luminescent nanostructure and offer great opportunities in the fields of optical applications.

## Introduction

In the last decade, lanthanide-doped upconversion nanoparticles have been widely studied because of their unique optical properties including narrow emission bandwidth, large Stokes shift, long luminescence lifetime and high photostability (Auzel, 2004; Lu et al., 2013; Bettinelli et al., 2015; Li et al., 2015, 2017; Jalani et al., 2018; Liu et al., 2018; Wang et al., 2018). These nanoparticles have the potential to be used in diverse applications such as biomedicine, data storage, solar energy conversion (Chen et al., 2015, 2019; Tsang et al., 2015; Zhou B. et al., 2015; Qi et al., 2017; Su et al., 2017; Zhu et al., 2017; Chen B. et al., 2018; Chen S. et al., 2018; Gai et al., 2018; Zheng et al., 2018; Ma et al., 2019). Particularly, the growing demand of lanthanide-doped nanoparticles using in various applications has in turn greatly stimulated basic research to develop novel nanoparticles with controlled size, shape, phase and desired properties (Wang et al., 2010, 2019; Du et al., 2016; Liu D. et al., 2016; Shi et al., 2017; Kang et al., 2019; Sun et al., 2019; Wang, 2019; Wu et al., 2019; Zhao et al., 2019; Zheng et al., 2019; Chen and Wang, 2020).

The assemblies of colloidal nanoparticles with unique structure and optical properties have considerable promise in various applications (Nie et al., 2010; Singamaneni et al., 2011; Boles et al., 2016; Ariga et al., 2019; Grzelczak et al., 2019; Runowski et al., 2019). However, the assembly of isotropic nanocrystals into anisotropic structures is a fundamental challenge in nanochemistry (Liu et al., 2010; Chen and Wang, 2019). Methods have been developed for organizing inorganic nanomaterials based on inherent anisotropy of magnetic (Zhang and Wang, 2008) or electric dipoles (Si et al., 2007), external magnetic (Hu et al., 2011) or electric field-induction (Rozynek et al., 2017), spatial confinement using hard or soft templates. Specific examples of templates include linear biomacromolecules (Braun et al., 1998; Tseng et al., 2006), block copolymers (Li et al., 2011; Kim et al., 2020), carbon nanotubes (Wang et al., 2006), and so on. Despite the considerable progress made in the past several years, self-assembly of nanoparticles into an anisotropic structure is still a daunting challenge because subtle variations in interparticle interactions can cause prominent morphology changes (Su et al., 2018). Moreover, anisotropic upconversion nanoparticle self-assemblies have been rarely reported (Liu X. et al., 2016; Ren et al., 2018; Yuan et al., 2018).

**Scheme 1:**
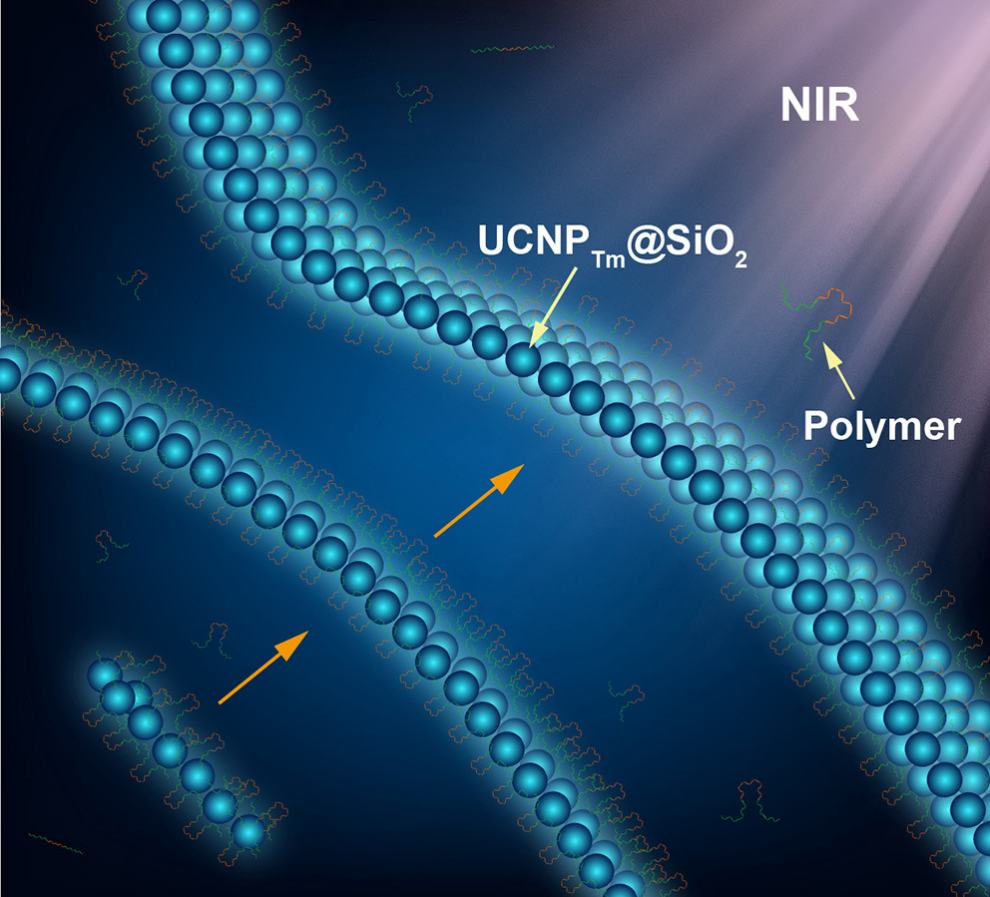
Schematic illustration of amphiphilic copolymer mediated self-assembly of silica-coated upconversion nanoparticles.

Here, we report a self-assembly method of upconversion nanoparticles mediated by amphiphilic block copolymer (Scheme 1). This approach can obtain chain-like assemblies that span multiple length scales from nanometers to micrometers. Our study reveals a time-dependent chain-like self-assembly process. Besides, we observe Y-junction aggregate morphology due to the flexible nature of amphiphilic block copolymer. Importantly, this study provides a new route to prepare anisotropic structures materials and offer exciting opportunities for optical applications.

## Materials and Methods

### Materials

Yttrium(III) acetate hydrate (99.9%), ytterbium(III) acetate hydrate (99.9%), thulium(III) acetate hydrate (99.9%), oleic acid (technical grade, 90%), 1-octadecene (technical grade, 90%), Igepal CO-520, tetraethyl orthosilicate (TEOS, >99.0%), ammonium fluoride (98%) sodium hydroxide (>98%), and Pluronic F127 were purchased from Sigma-Aldrich. Methanol (99.5%), cyclohexane (analytical grade), and ammonia solution (25–28%) were obtained from Aladdin. All chemicals were used as received without further purification.

### Characterization

Low-resolution transmission electron microscopy (TEM) measurements were carried out on a JEOL-JEM 2010F field emission transmission electron microscopy operated at an acceleration voltage of 200 kV. Powder X-ray diffraction (XRD) data were recorded on a Bruker D8 Advance diffractometer with a graphite monochromatized CuKα radiation (1.5406 Å). Luminescence spectra were recorded at room temperature with a DM150i monochromator equipped with an R928 photon-counting photomultiplier tube (PMT), in conjunction with a 980-nm diode laser. Upconversion luminescence microscopy imaging was performed on an Olympus BX51 microscope with the xenon lamp adapted to a diode laser. Luminescence micrographs were recorded with a Nikon DS-Ri1 imaging system. Digital photographs were taken with a Nikon D700 camera.

### Synthesis of NaYF_4_:Yb,Tm Nanoparticles

In a typical procedure, a solution of Ln(CH_3_CO_2_)_3_ (0.2 M, Ln = Y, Yb, Tm) in water (2 mL), and oleic acid (4 mL) were added to a 50 mL two-neck flask and then heated to 150°C for 30 min to remove the water content from the mixture. Then 1-octadecene (6 mL) was quickly added to the flask and the resulting mixture was maintained at 150°C for another 30 min before cooling down to 50°C. Shortly thereafter, 5 mL of a methanol solution containing NH_4_F (1.36 mmol) and NaOH (1 mmol) was added and the resultant mixture was stirred for 30 min at this temperature. After the methanol was evaporated, the solution was heated to 300°C under argon for 1.5 h and then cooled down to room temperature. The resulting nanoparticles were precipitated by the addition of excess ethanol, collected by centrifugation at 6,000 rpm for 5 min, and washed with ethanol several times before dispersing them in 2 mL of cyclohexane for optical and TEM measurements.

### Synthesis of NaYF_4_:Yb,TmNaYF_4_ (UCNP_Tm_) Nanoparticles

The as-synthesized NaYF_4_:Yb,Tm core nanoparticles were used as seeds to epitaxial overgrowth of NaYF_4_ layer. For the preparation of shell precursors, 0.4 mmol of Y(CH_3_CO_2_)_3_ was used. The synthetic procedure is similar to the synthesis of core nanoparticles.

### Preparation of Hydrophilic Ligand-Free UCNP_Tm_ Nanoparticles

Ligand-free nanoparticles were prepared following a literature procedure (Su et al., 2012). The as-synthesized oleic acid-capped UCNP_Tm_ nanoparticles were dispersed in a mixed solution of HCl (1 mL; 0.2 M) and ethanol (1 mL) and then sonicated for 5 min to remove the surface-capped ligands. The resulting ligand-free nanoparticles were centrifuged at 16,500 rpm for 20 min. The products were finally washed with ethanol and DI water several times and then re-dispersed in DI water.

### Synthesis of NaYF_4_:Yb,Tm@NaYF_4_@SiO_2_ (UCNP_Tm_@SiO_2_) Nanoparticles

The synthesis of silica-coated UCNP_Tm_ nanoparticles was carried out following a literature procedure (Han et al., 2017). One milliliter of Igepal CO-520 was mixed with 20 mL cyclohexane in a flask and stirred for 1 h. As-synthesized ligand-free UCNP_Tm_ nanoparticles (1.5 mL) was then added into the mixture and stirred for 3 h at room temperature. After that, NH_3_·H_2_O (150 μL, 30%) was added into the resulting mixture and stirred for another 2 h. A solution composed of TEOS (0.2 mL) and cyclohexane (0.8 mL) was introduced into the flask within 1 h by using a syringe pump. Subsequently, the mixture was hermetically stirred for 24 h at room temperature. The as-prepared products were precipitated by methanol and then centrifuged at 12,000 rpm for 10 min. The nanoparticles were then washed with a mixture of ethanol and cyclohexane three times. Finally, the nanoparticles were dispersed in deionized water.

### Self-Assembly of Upconversion Nanoparticles by F127

In a typical experiment, 100 μL UCNP_Tm_@SiO_2_ aqueous solution (1 mg mL^−1^) was diluted in 3 mL DI water. 0.6 mg F127 was added to the solution and then heated to 60°C. After stirring for 1 h, the resultant suspension was incubated at 60°C for a certain time (12, 24, 48 h, and 7 days) without agitation. A few drops of nanoparticle solution were dropped onto the glass slide. Subsequently, the luminescence micrographs were captured under a recorded with a Nikon DS-Ri1 color imaging system.

## Results and Discussion

### Characterization of Upconversion Nanoparticles

In terms of efficient upconversion luminescence, the inherent property of host materials and crystal nanostructure play key roles (Chen et al., 2014; Zhou J. et al., 2015). Hexagonal NaYF_4_ is regarded as ideal host materials because of the low phonon energy and high photochemical stability (Wang et al., 2010). Additionally, optical inert NaYF_4_ host materials can avoid unwanted energy consumption, i.e., surface quenching of the fluorescence (Su et al., 2012). Therefore, Hexagonal NaYF_4_ was chosen as the host matrix to obtain efficient upconversion luminescence. To realize good water solubility, we coated a layer of SiO_2_ onto the surface of upconversion nanoparticles and use them as an experimental model for the self-assembly demonstration.

**Figure 1:**
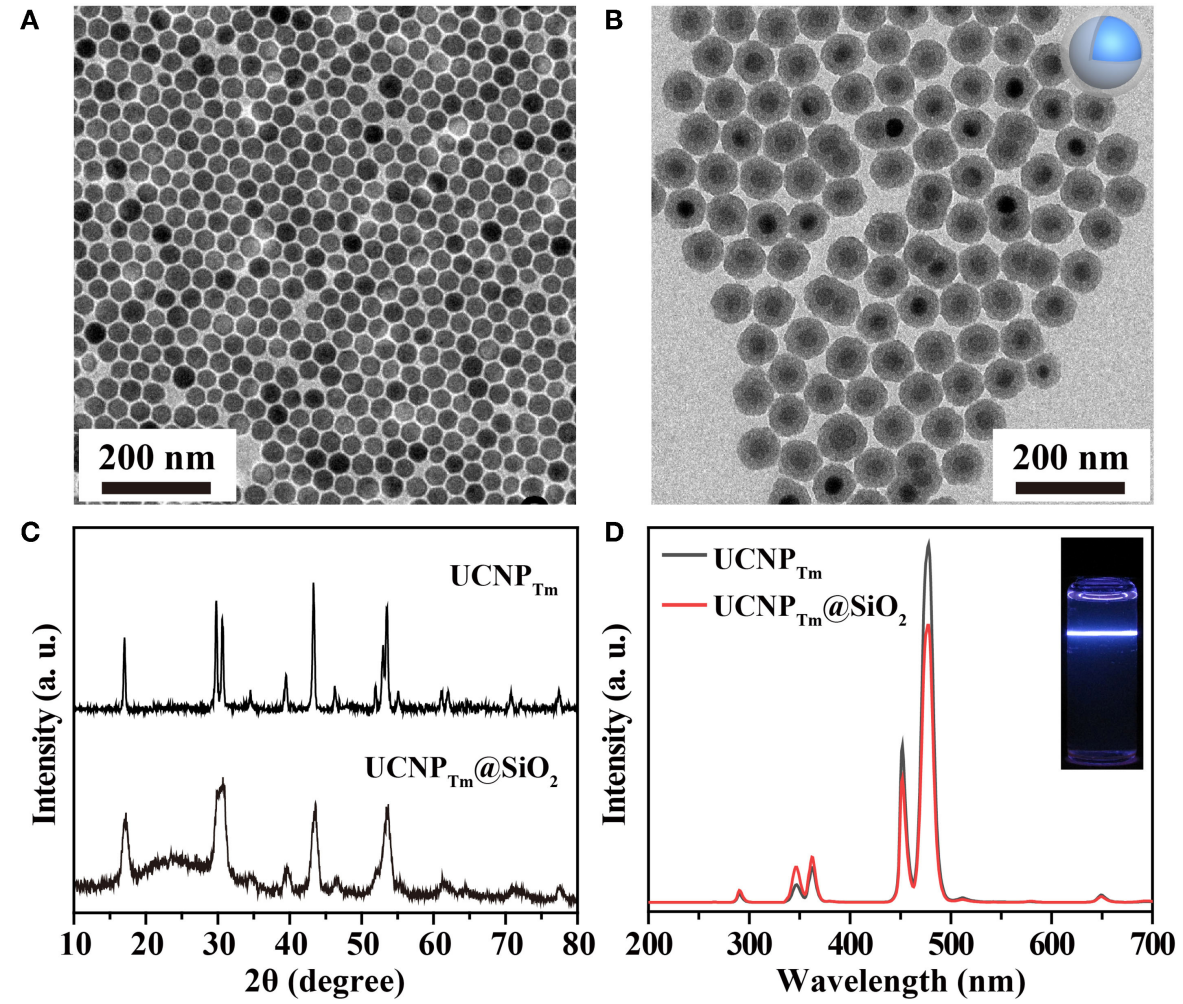
**(A)** TEM image of NaYF_4_:Yb,Tm nanoparticles in cyclohexane. **(B)** TEM image of NaYF_4_:Yb,Tm@SiO_2_ nanoparticles in water. **(C)** XRD patterns of NaYF_4_:Yb,Tm and NaYF_4_:Yb,Tm@SiO_2_ nanoparticles. **(D)** The corresponding emission spectrum of the as-prepared nanoparticles and their silica-coated counterpart. Inset: luminescence photograph of SiO_2_ coated-nanoparticles in water under irradiation of a 980 nm laser.

We began with the synthesis of oleic acid-capped NaYF_4_:Yb,Tm@NaYF_4_ (UCNP_Tm_) and NaYF_4_:Yb,Er@NaYF_4_ (UCNP_Er_) core-shell nanospheres through an epitaxial growth method (Abel et al., 2009; Wang and Chen, 2019). The as-synthesized nanospheres were characterized using a transmission electron microscope (TEM). Uniform UCNP_Tm_ nanoparticles with an average diameter of about 36 nm for UCNP_Tm_ were obtained and shown in Figure 1A and Supplementary Figure 1. After silica coating on the surface of UCNP_Tm_ nanospheres, the size of the nanoparticles reached about 76 nm (Figure 1B and Supplementary Figure 2). The dynamic light scattering (DLS) measurement show that the hydrodiameter of UCNP_Tm_@SiO_2_ nanoparticles was around 90 nm (PDI = 0.13), demonstrating their mono-dispersion in water (Supplementary Figure 3). X-ray powder diffraction (XRD) analysis was conducted to confirm the phase-purity of UCNP_Tm_ nanoparticles, which can be indexed as a hexagonal phase of NaYF_4_ (JCPDS file number 16-0334) (Figure 1C). A broad diffraction peak at 2θ = 22° appeared in UCNP_Tm_@SiO_2_ nanoparticles pattern, which can be ascribed to the peak of amorphous silica (Zhou et al., 2019).

We next studied the optical properties of as-prepared upconversion nanoparticles. Upon 980 nm excitation, Tm^3+^ ions in UCNP_Tm_ nanoparticles exhibit a characteristic emission at 290 nm (^1^I_6_ → ^3^H_6_), 345 nm (^1^I_6_ → ^3^H_5_), 360 nm (^1^D_2_ → ^3^H_6_), 450 nm (^1^D_2_ → ^3^F_4_), 475 nm (^1^G_4_ → ^3^H_6_), 511 nm (^1^D_2_ → ^3^H_5_) and 650 nm (^1^G_4_ → ^3^F_4_) from ultraviolent to visible region (Wang and Liu, 2008), respectively (Figure 1D). After silica coating, the luminescent intensity was slightly weaker compared to oleic acid-coated nanoparticles. The resulting nanoparticles were dispersed in DI water prior to being used for self-assembly demonstration (Figure 1D, inset).

**Figure 2:**
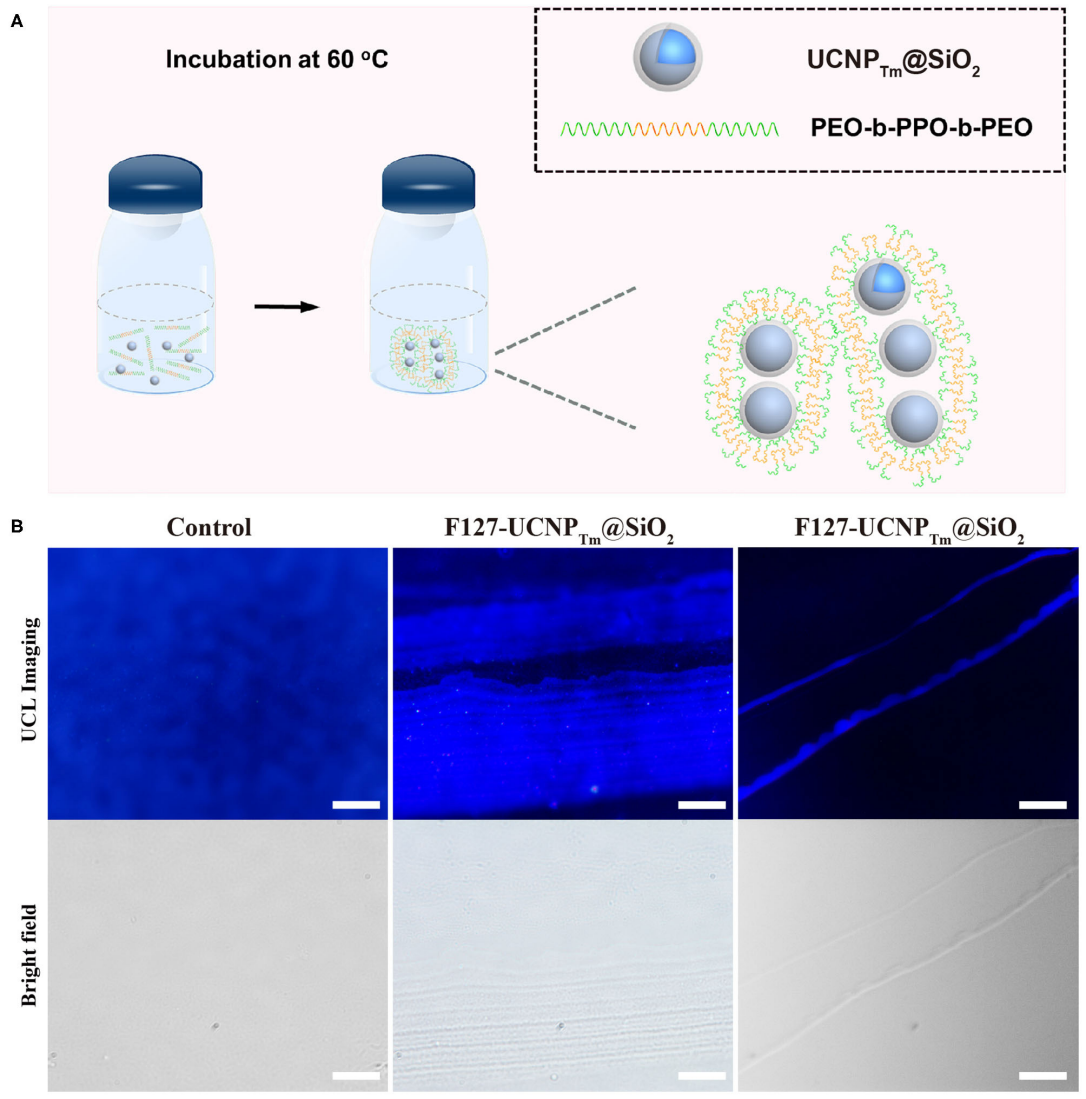
**(A)** Schematic diagram of the amphiphilic copolymer mediated self-assembly process. **(B)** Luminescence micrograph of UCNP_Tm_@SiO_2_ nanoparticles incubated with and without F127 mediation for 7 days. The scale bars are 10 μm (left and middle column) and 100 μm (right column), respectively.

As a proof-of-concept experiment, the amphiphilic copolymer F127 was employed as a surfactant to mediate the self-assembly process of UCNP_Tm_@SiO_2_ nanoparticles. The procedure the UCNP_Tm_@SiO_2_ nanoparticles self-assembly was shown in Figure 2A. In a typical experiment, we simply mixed UCNP_Tm_@SiO_2_ and amphiphilic copolymer F127 in aqueous solution and then heated to 60°C. Upon aging the nanoparticles in amphiphilic copolymer aqueous solution for 7 days without agitation, upconversion nanoparticles long belts gradually generated. As shown in Figure 2B (middle and right column), long upconversion nanoparticles belts composed by horizontally arranged nanoparticle chains were clearly observed and the nanoparticle belts reach to micro-scale. However, we didn't observe any organized structure without the addition of copolymer F127 (Figure 2B, left column). To our knowledge, this is the first observation of a micrometer scale upconversion nanoparticles belts by solution self-assembly of amphiphilic block copolymers.

**Figure 3:**
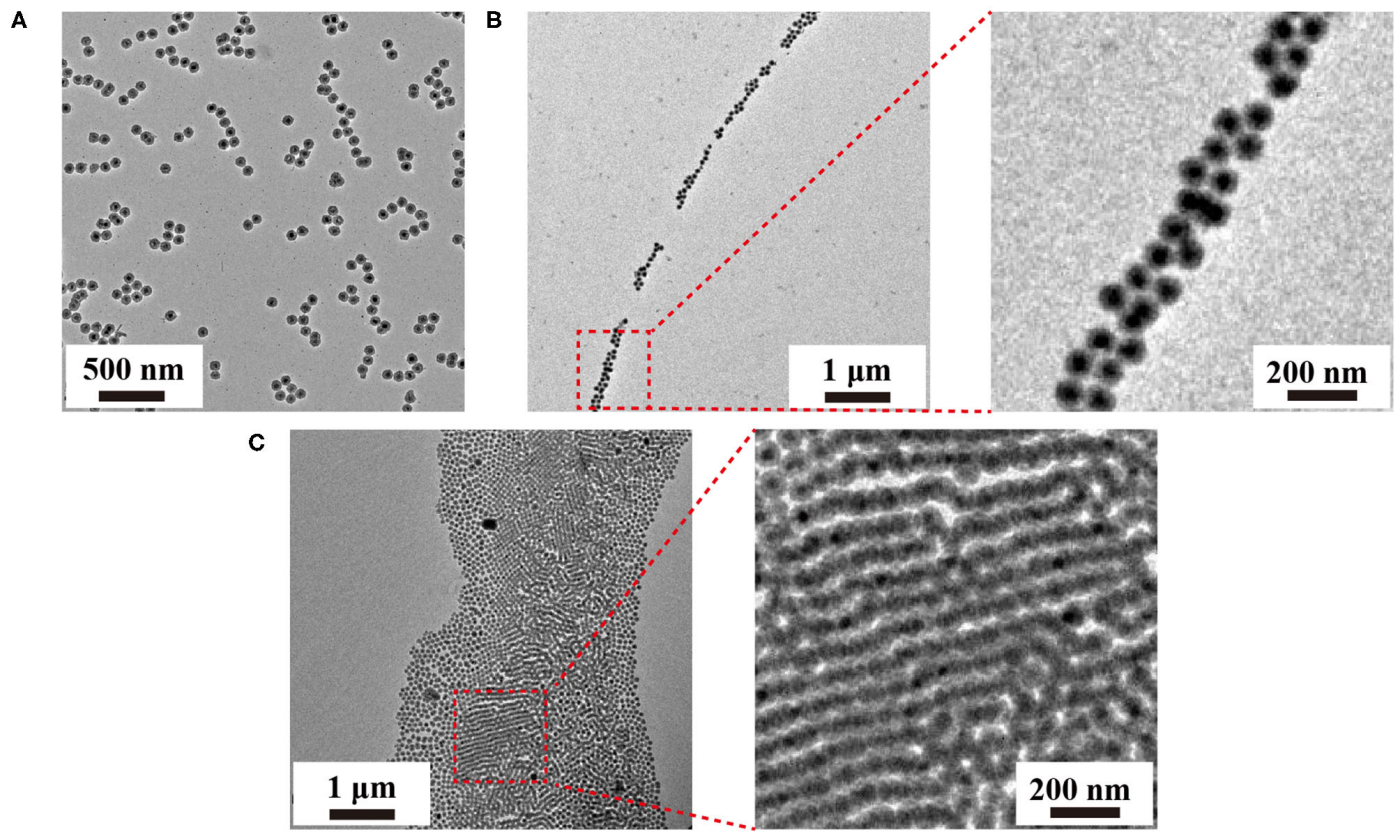
TEM images of the as-prepared upconversion nanoparticle assemblies mediated by F127 under varied incubation time, 12 h **(A)**, 24 h, **(B)**, 48 h **(C)**, respectively.

The formation of the upconversion nanoparticle chains was then studied using TEM as shown in Figure 3A. The nanoparticles connected in a one-dimensional tendency upon short incubation time (12 h) with amphiphilic copolymer F127. TEM images revealed the presence of the short chains composed by several to a dozen nanoparticles. The interparticle separations between UCNP_Tm_@SiO_2_ nanoparticles in the chain were observed (Supplementary Figure 4), indicating that the obtained nanoparticle chains are connected by the molecular linker. Noted that hot water can etch away the SiO_2_ shell by breaking the internal Si-O-Si bonds (Liu et al., 2014). Our nanoparticles were slightly etched due to the protection of F127 copolymer. When the incubation time was prolonged to 24 h, we observed long chains of UCNP_Tm_@SiO_2_ nanoparticles which reached microscale in length (Figure 3B). The nanoparticles chains have an average width of 1–3 nanoparticles. Interestingly, further prolonging the incubation time resulted in the formation of upconversion nanoparticle belts. As shown in Figure 3C, TEM images revealed the presence of the belts with an average belt length of 40–50 nanoparticles and as wide as about 2.5–4 μm. Furthermore, these nanoparticles under the drive of interfacial energy would spontaneously assemble in close-packed arrangements during the extended incubation period. This result is also consistent with the luminescence micrographs of UCNP_Tm_@SiO_2_ nanoparticle showing horizontally arranged nanoparticle chains. By contrast, we observe a random pattern of UCNP_Tm_@SiO_2_ nanoparticles without the addition of copolymer F127 (Supplementary Figure 5).

**Figure 4:**
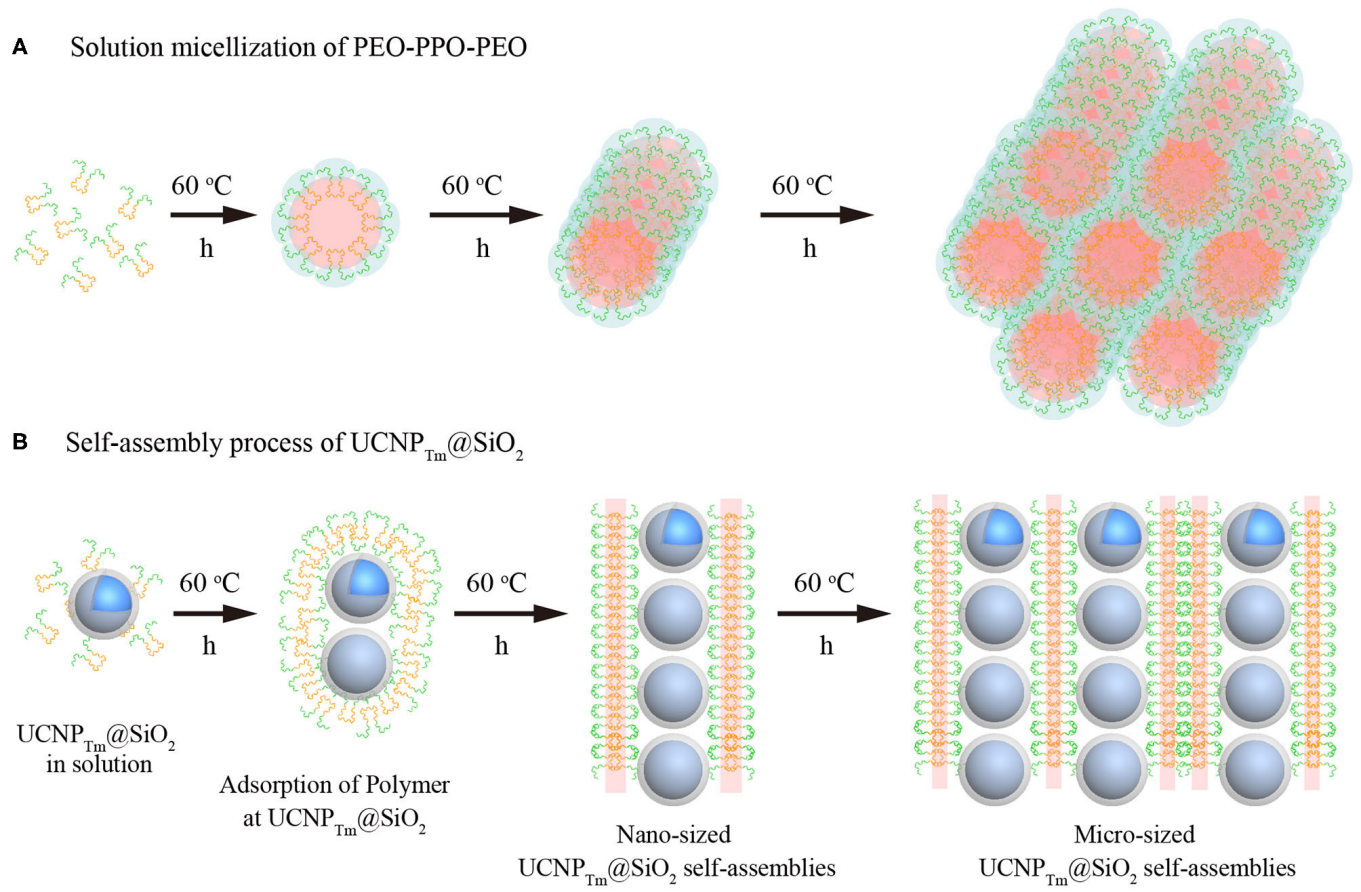
**(A)** Morphological transitions of amphiphilic block copolymers F127 as a function of time. **(B)** Schematic illustration of the self-assembly process for SiO_2_-coated upconversion nanoparticles. Note that the green chain denotes hydrophilic segment PEO, and the orange chain represents hydrophobic segment PPO of F127, respectively.

The ability of amphiphilic block copolymers to drive UCNP_Tm_@SiO_2_ nanoparticles self-assemble into chain-like nanostructures when dissolved in water is based on the specific amphiphilic character of this block copolymer F127. These amphiphilic block copolymers consist of a polar, hydrophilic polymer block (PEO) and a non-polar, lipophilic polymer block (PPO). In water, which is a thermodynamically good solvent for the PEO block but a poor solvent for the PPO block, the “insoluble” PPO block aggregates and forms a core while the “soluble” PEO block forms a corona that interacts with the water and stabilizes the polymer self-assembly (Alexandridis et al., 1994; Liu and Li, 2015). As shown in Figure 4A, the formation of one-dimensional chains may be induced by the transition from spherical to long cylindrical micelles of the amphiphilic block copolymer. It should be noted that the self-assembly of amphiphilic block copolymers in specific block-selective solvents generates morphologies such as spheres, cylindrical micelles, and a variety of other architectures (Kang et al., 2005; Gilroy et al., 2010).

SiO_2_ coated upconversion nanoparticles exhibited durable super-hydrophilic surface properties due to that the surface of silica particles is covered with SiOH groups. The PEO-PPO-PEO block copolymer can be absorbed on the hydrophilic surfaces. On hydrophilic surfaces, these block copolymer surfactants adsorb with their hydrophilic tail (PEO) toward the SiO_2_ surface, resulting in double-layer adsorption (Malmsten et al., 1992). Furthermore, when the incubation time was prolonged, the size of the cylindrical micelles became larger in width and length, and thus micro-scaled nanoparticle assembly was formed (Figure 4B). It should be noted that our results are also consistent with the previous experimental evidence showing that the length of cylindrical block copolymer micelles could reach up to ~ 200 nm to 2 μm (Gilroy et al., 2010). Taken together, we suggest that UCNP_Tm_@SiO_2_ nanoparticles prefer to assemble in a chain manner might be caused by the mutual attraction between the hydrophilic-hydrophilic interactions (Fukao et al., 2009) and cylindrical micelles formed by the self-assembly process of block copolymers F127.

**Figure 5:**
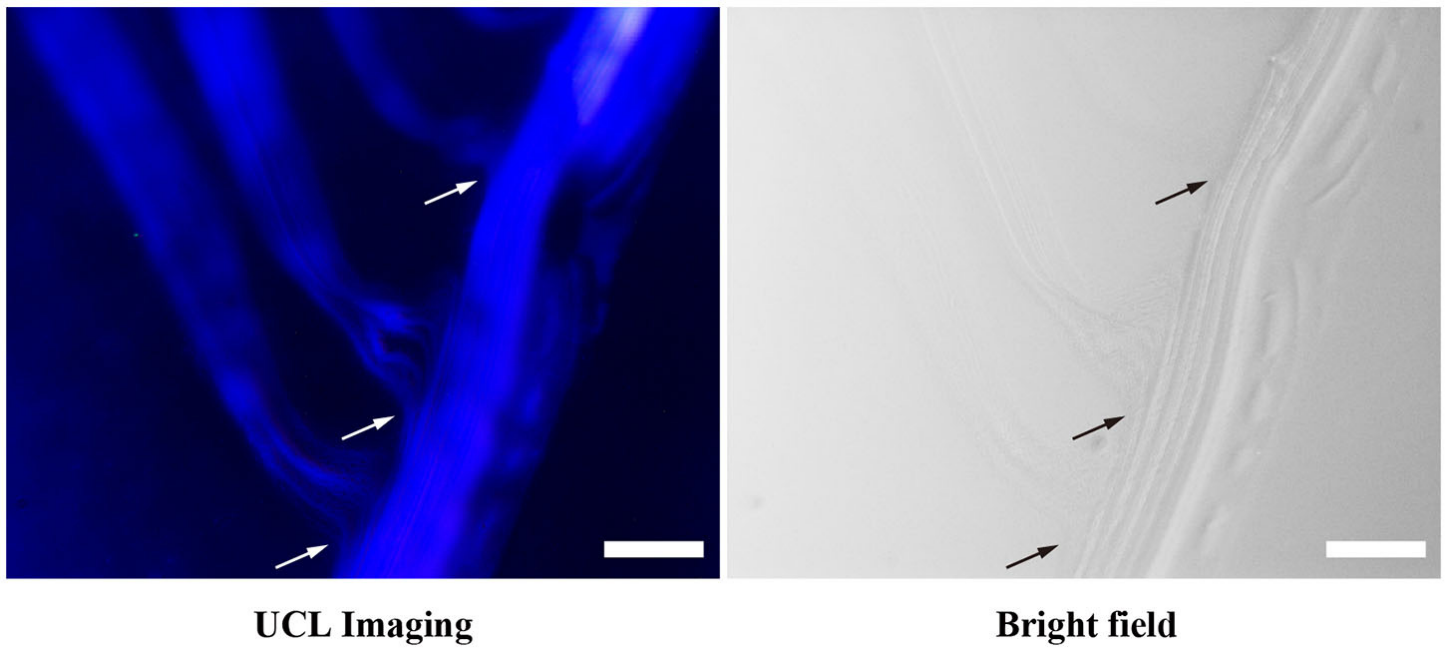
Luminescence micrograph demonstrates the existence of Y-junctions of UCNP_Tm_@*SiO*_2_ assemblies incubated with F127 mediation for 7 days. Arrows indicate examples of Y-junctions. The scale bars are 10 μm.

**Figure 6:**
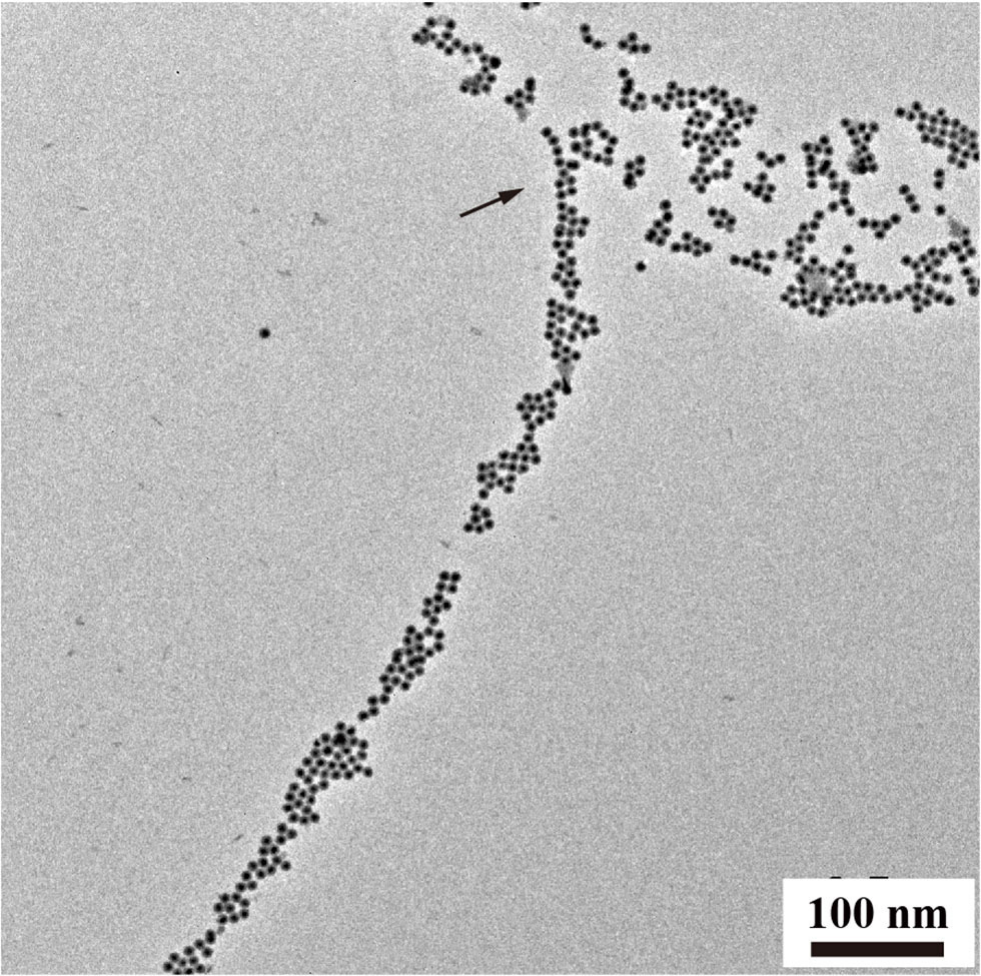
TEM analysis of Y-junctions of UCNP_Tm_@*SiO*_2_ assemblies incubated with F127 mediation for 24 h. Arrows indicate examples of Y-junctions.

In addition to chain-like assemblies spanning from the nanometer to the micrometer length scale, we also observed occasional Y-junctions of UCNP_Tm_@SiO_2_ assemblies incubated with F127 mediation for 7 days by using the luminescence microscope and the image was shown in Figure 5. To further demonstrate the existence of Y-junctions, we carefully examined the 24 h-incubation samples of UCNP_Tm_@SiO_2_ by TEM image. As expected, we observed the formation of Y-junctions, and TEM images were shown in Figure 6. This morphology may be ascribed to typical structural defects associated with the formation of the cylindrical micelle (Fenyves et al., 2014). It was reported that the formation of structural defects (i.e., Y-junctions) becomes prevalent when micellization kinetics slow down (Tlusty and Safran, 2000). We observed some Y-junctions (Supplementary Figure 6). We hypothesized that such a behavior can be ascribed to the flexible nature of F127 copolymer, which can accommodate packing frustrations in structures that have significant deviations from the mean curvature. This explanation was consistent with the previous report indicating that the packing frustrations associated with deviations from the mean curvature in the Y-junctions would be alleviated by chain stretching of polymers with high molecular weight (Jain and Bates, 2003). Moreover, our mechanism is different from the DNA mediated self-assembly mechanism reported by Jin and coworkers, which involves anisotropic surface functionalization of upconversion nanoparticle that directs the anisotropic pattern of upconversion nanoparticle self-assembly (Ren et al., 2018).

## Conclusions

In conclusion, we have presented a novel approach for controlling the one-dimensional assemblies of upconversion nanoparticles at different scales from nano-scale to micro-scale. The results revealed time-dependent morphology, producing chain-like and belt-like structures. Our study suggests that the morphology is depending on the morphology of micelles of amphiphilic block copolymers in specific conditions. And the driving force for the self-assembly of upconversion nanoparticles is particle surface hydrophobic and hydrophilic force offered by amphiphilic polymers. Furthermore, this work may lead to new applications for upconversion nanoparticles and may provide insightful ideas for the design of other functional nanomaterials.

## Data Availability Statement

All datasets generated for this study are included in the article/Supplementary Material.

## Author Contributions

QSu and YS conceived the project. QSu, M-TZ, YS, M-ZZ, and QSun were primarily responsible for the experiments of nanoparticle synthesis and characterization. QSu, M-TZ, YS, and TA contributed to the data analyses and discussion. QSu, YS, and TA prepared figures and wrote the paper with input from other authors. All authors contributed to the article and approved the submitted version.

## Conflict of Interest

The authors declare that the research was conducted in the absence of any commercial or financial relationships that could be construed as a potential conflict of interest.
