# Supplementary material for: Microscale Self-Assembly of Upconversion Nanoparticles Driven by Block Copolymer
Source: Front Chem. 2020 Sep 16;8:836. doi: 10.3389/fchem.2020.00836 (PMC7528114; doi:10.3389/fchem.2020.00836)
Supplement: Supplementary file 1 [file Data_Sheet_1.docx]

Supplementary Material

Microscale Self-assembly of Upconversion Nanoparticles Driven by Block Copolymer

Qianqian Su^1,2,#,^*, Meng-Tao Zhou^1,#^, Ming-Zhu Zhou^1^, Qiang Sun^3^, Taotao Ai^4^, Yan Su^5,^*

^1^Institute of Nanochemistry and Nanobiology, Shanghai University, Shanghai 200444, China

^2^Department of Chemistry, National University of Singapore, Singapore 117543, Singapore

^3^Center for Functional Materials, NUS (Suzhou) Research Institute, Suzhou, Jiangsu 215123, China

^4^National and Local Joint Engineering Laboratory for Slag Comprehensive Utilization and Environmental Technology, School of Materials Science and Engineering, Shaanxi University of Technology, Hanzhong, Shaanxi 723000, China

^5^Genome Institute of Singapore, Agency of Science Technology and Research, 138672, Singapore

***Correspondence:** Corresponding Authors: chmsqq@shu.edu.cn (Q. Su) and suy1@gis.a-star.edu.sg (Y. Su)

^#^These authors contributed equally.

**
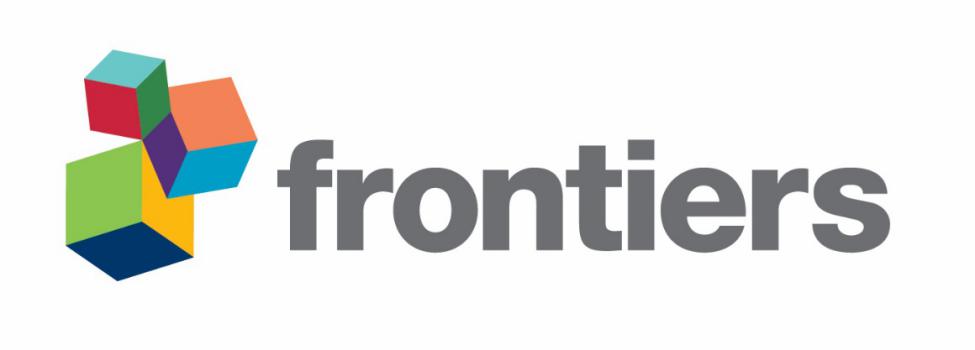
**

**
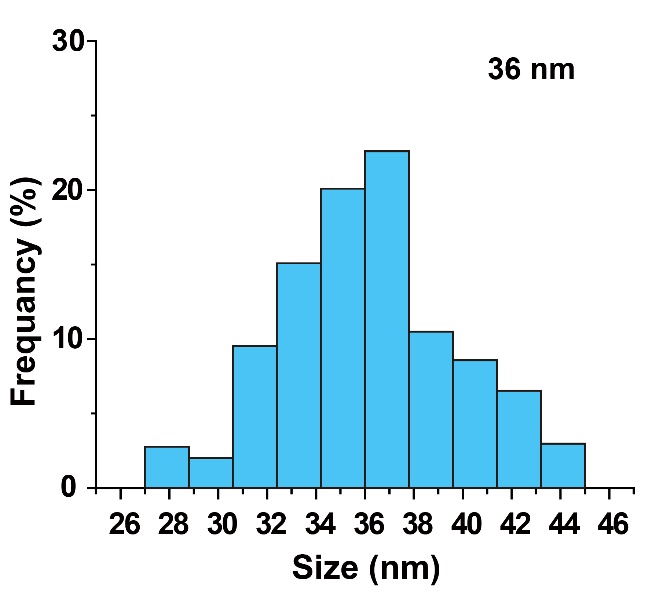
**

**Figure S1.** Size distribution of NaYF_4_:Yb,Tm@NaYF_4_ nanoparticles.


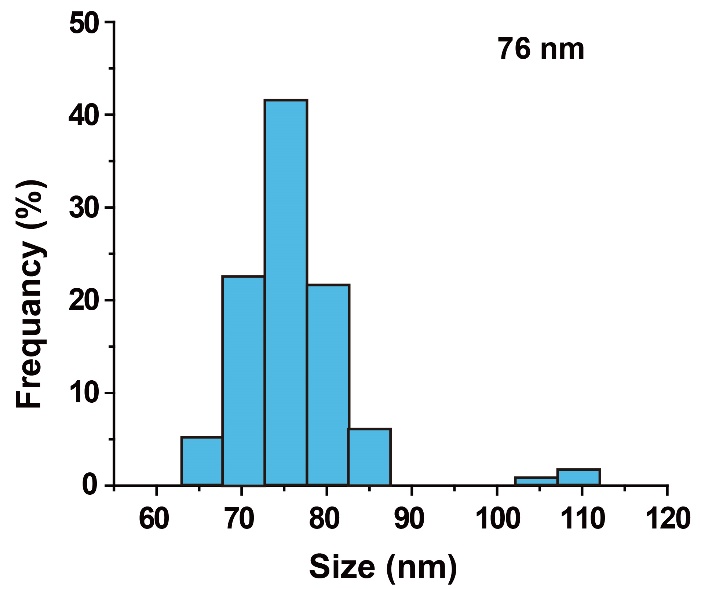


**Figure S2.** Size distribution of NaYF_4_:Yb,Tm@NaYF_4_@SiO_2_ (UCNP_Tm_@SiO_2_) nanoparticles.


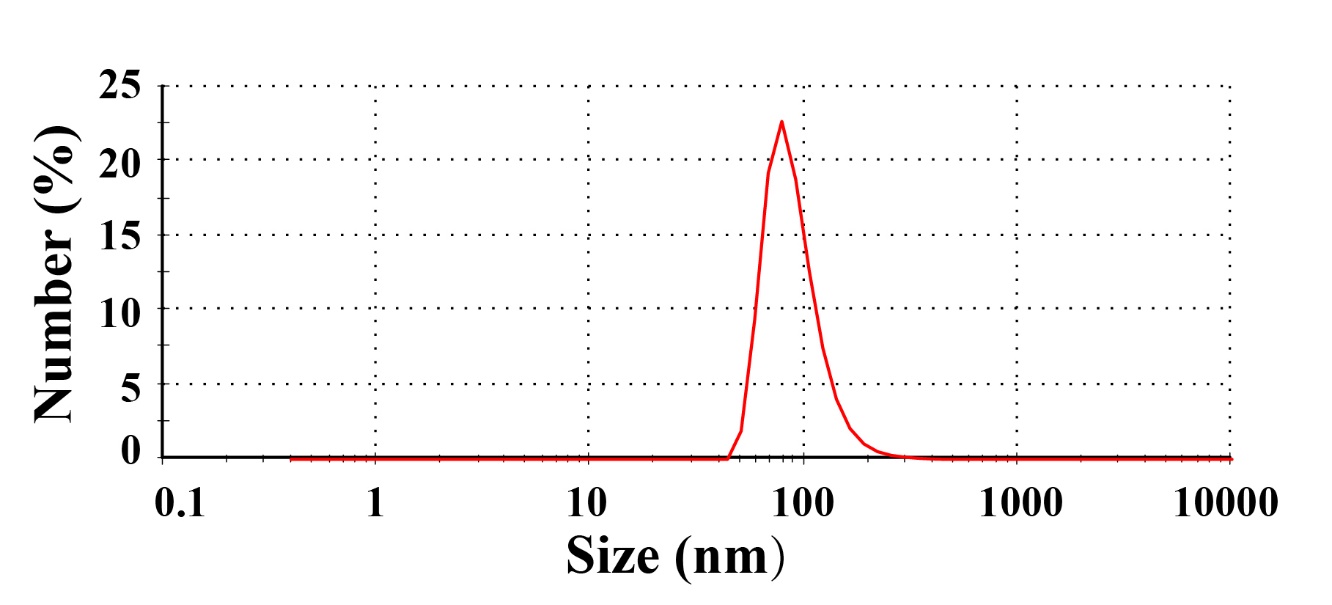


**Figure S3.** Dynamic light scattering (DLS) of UCNP_Tm_@SiO_2_ nanoparticles in water.


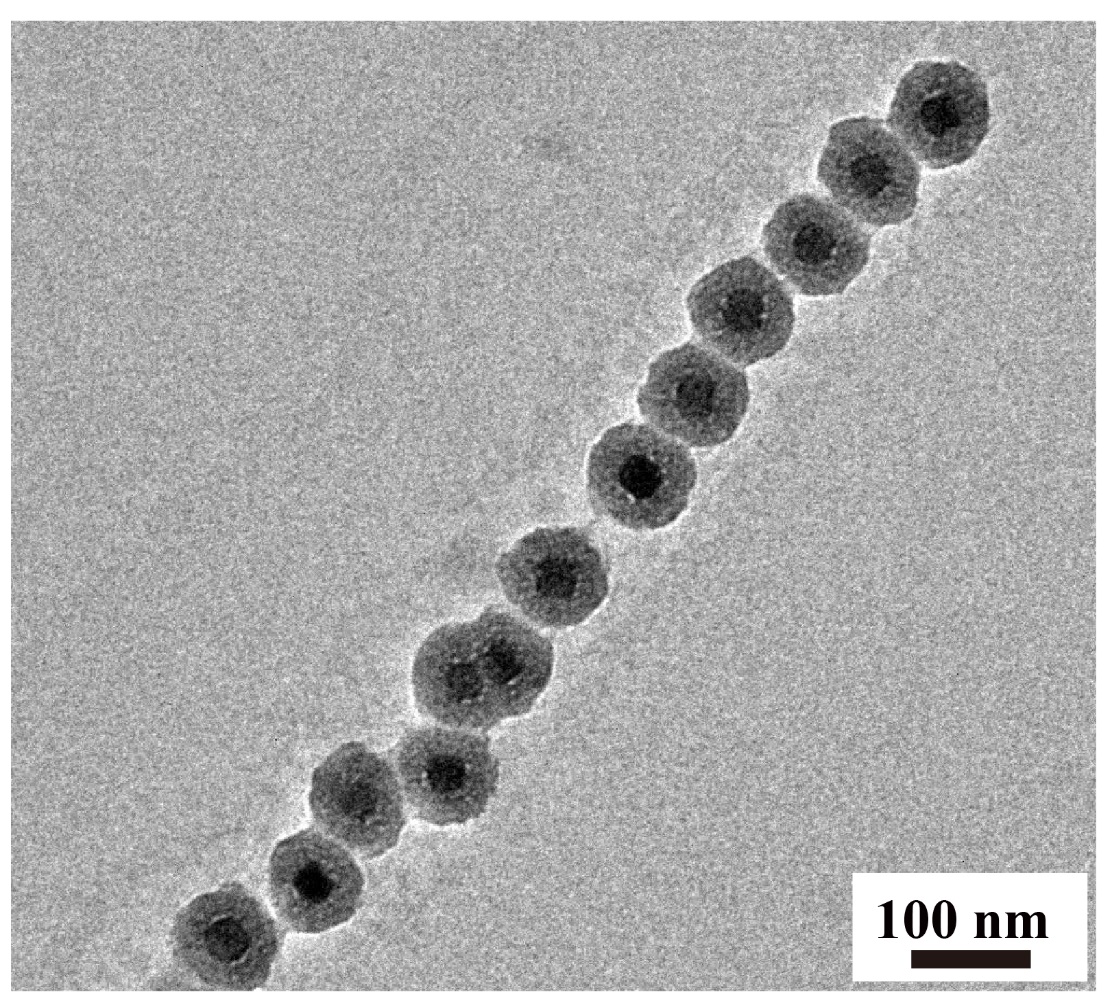


**Figure S4.** TEM images of the as-prepared UCNP_Tm_@SiO_2_ nanoparticle chain mediated by F127 for 12 hours.

**
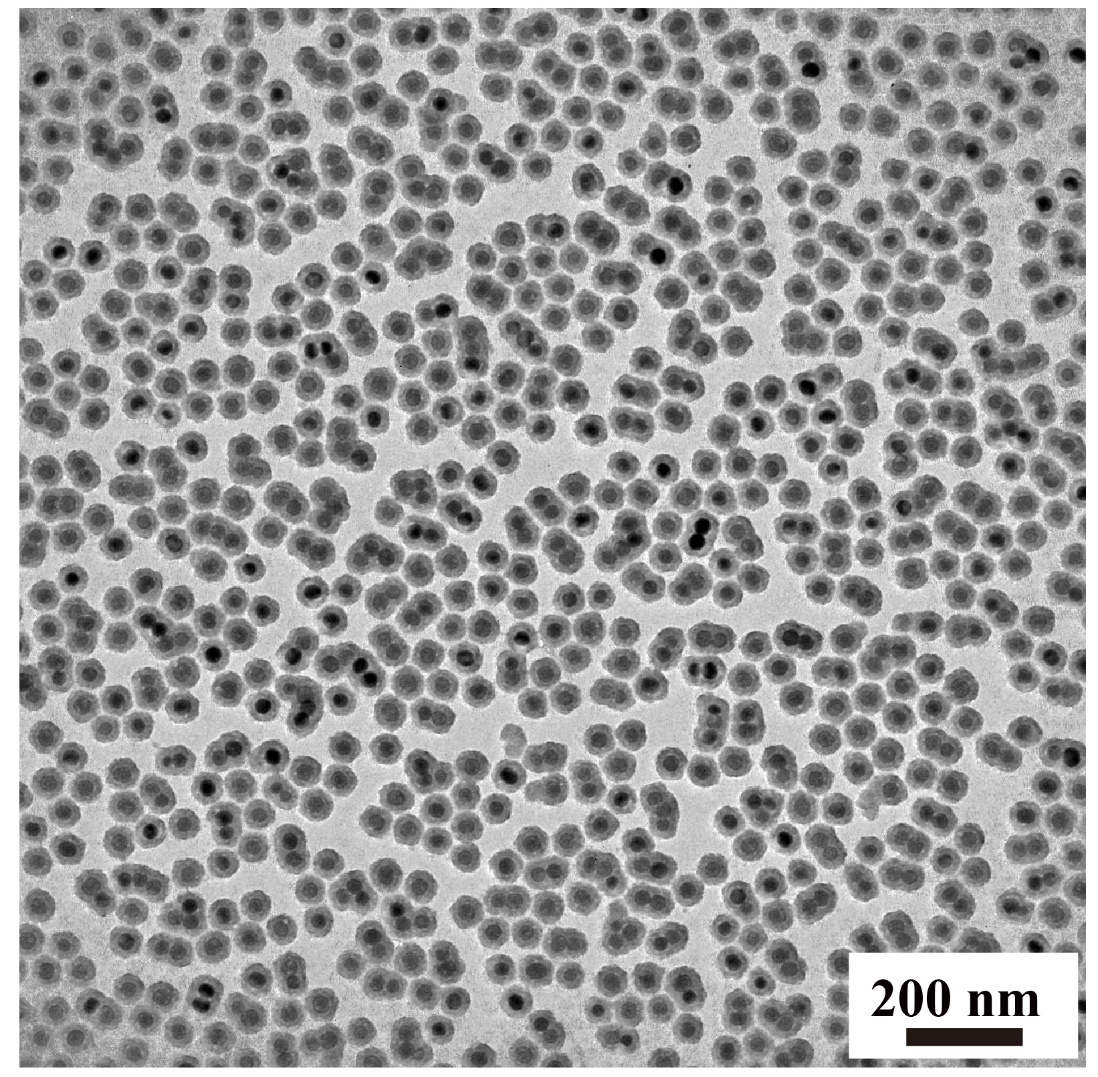
**

**Figure S5.** TEM images of the as-prepared UCNP_Tm_@SiO_2_ nanoparticles incubated in water without F127.

**
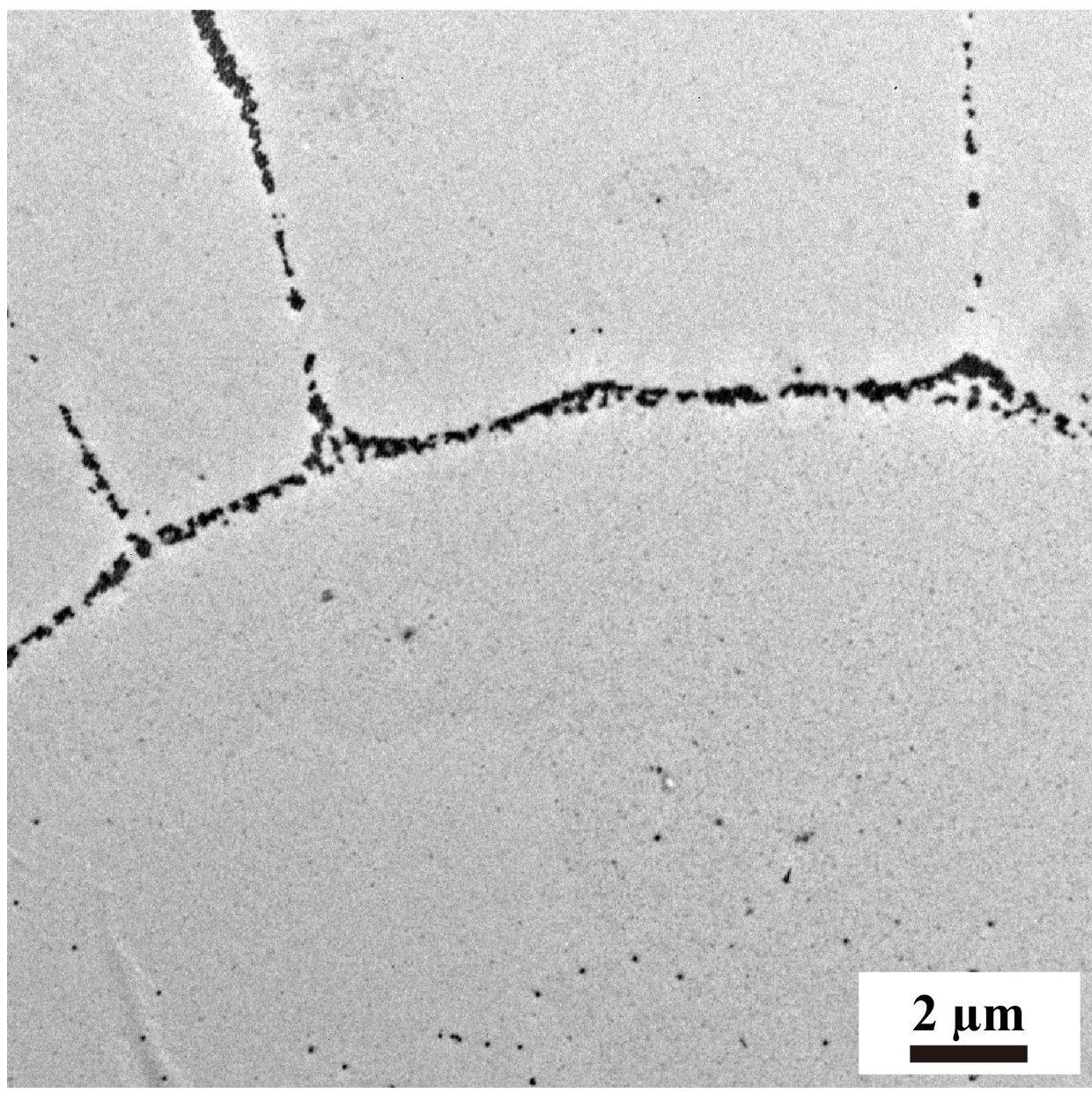
**

**Figure S6.** TEM demonstrated the existence of Y-junctions of UCNP_Tm_@SiO_2_ assemblies incubated with F127 mediation for 24 hours.
